# Complicated Postoperative Course after Pulmonary Artery Sling Repair and Slide Tracheoplasty

**DOI:** 10.3389/fped.2017.00067

**Published:** 2017-04-10

**Authors:** Angelika Weber, Birgit Donner, Marie-Hélène Perez, Stefano Di Bernardo, Daniel Trachsel, Kishore Sandu, Nicole Sekarski

**Affiliations:** ^1^Pediatric Cardiology Unit, Department of Pediatrics and Pediatric Surgery, University Hospital Lausanne, Lausanne, Switzerland; ^2^Division of Pediatric Cardiology, University Children’s Hospital Basel, Basel, Switzerland; ^3^Pediatric Intensive Care Unit, Department of Pediatrics and Pediatric Surgery, University Hospital Lausanne, Lausanne, Switzerland; ^4^Division of Pediatric Pulmonology, University Children’s Hospital Basel, Basel, Switzerland; ^5^Airway Unit, Service of Otorhinolaryngology, University Hospital Lausanne, Lausanne, Switzerland

**Keywords:** pulmonary artery sling, slide tracheoplasty, pulmonary artery thrombosis, pediatric cardiology, pediatric intensive care

## Abstract

Pulmonary artery sling (PAS) is a rare congenital condition in which the left pulmonary artery (LPA) arises from the right pulmonary artery, and then passes between the trachea and the esophagus to reach the left lung, thereby forming a sling around the airway. It is often associated with intrinsic tracheal stenosis due to complete cartilaginous rings. Therapeutic management nowadays consists of one-stage reimplantation of the LPA and tracheoplasty with cardiopulmonary bypass support. Here, we present a 7-week-old boy with PAS and long-segment tracheal stenosis (LSTS) who underwent surgical intervention consisting of reimplantation of the LPA and slide tracheoplasty. Multiple respiratory and cardiovascular complications marked the postoperative course. They consisted of recurrent failed attempts in weaning off mechanical ventilation due to bronchomalacia, left vocal cord paralysis, development of granulation tissue at the anastomosis and restenosis of the trachea, and the main stem bronchi requiring balloon dilatation. The patient also developed bilateral pulmonary artery thrombosis and stenosis of the LPA. After a prolonged hospitalization, the patient is doing well without any respiratory symptoms and has a good result on follow-up bronchoscopy 1 year after the initial surgery. The stenosis of the LPA responded well to percutaneous balloon dilatation 12 months after the primary surgery. The case illustrates that even though surgical techniques are improving and are in general associated with a low morbidity and mortality, management of PAS and tracheal stenosis can still be challenging. However, good long-term outcome can be achieved if the initial postoperative phase is overcome.

## Introduction

Pulmonary artery sling (PAS) is a rare congenital condition in which the left pulmonary artery (LPA) presents an aberrant origin from the posterior aspect of the right pulmonary artery. The LPA passes between the trachea and the esophagus to the left lung, thereby forming a sling around the airway (1). It is often associated with a patent ductus arteriosus (PDA) (2), originating from the main pulmonary artery, which may contribute to the encirclement and compression of the trachea. In addition to the extrinsic obstruction, an intrinsic tracheal stenosis due to complete cartilaginous rings is often coexisting, which led to the introduction of the term “ring-sling complex” (3).

The condition usually presents in the first weeks or months of life with progressive respiratory symptoms (stridor, wheezing, and cyanosis) and depending on the severity of the airway stenosis there may be necessity of ventilator support (1). Patients with PAS and long-segment tracheal stenosis (LSTS) require surgical intervention in the first year of life, which consists of reimplantation of the LPA on the main pulmonary artery and concomitant tracheal reconstruction with cardiopulmonary bypass (4, 5). Postoperative morbidity is mostly determined by complications of the tracheoplasty, such as restenosis or growth of granulation tissue, or an underlying tracheobronchomalacia (5, 6).

We describe the case of an infant with PAS and LSTS who presented a complicated postoperative course, with bilateral pulmonary artery thrombosis, stenosis of the LPA, tracheal restenosis, and vocal cord paralysis. The parents gave their written informed consent to the publication of this case, and it was approved by our institutional Ethics Committee.

## Case Report

**Figure 1:**
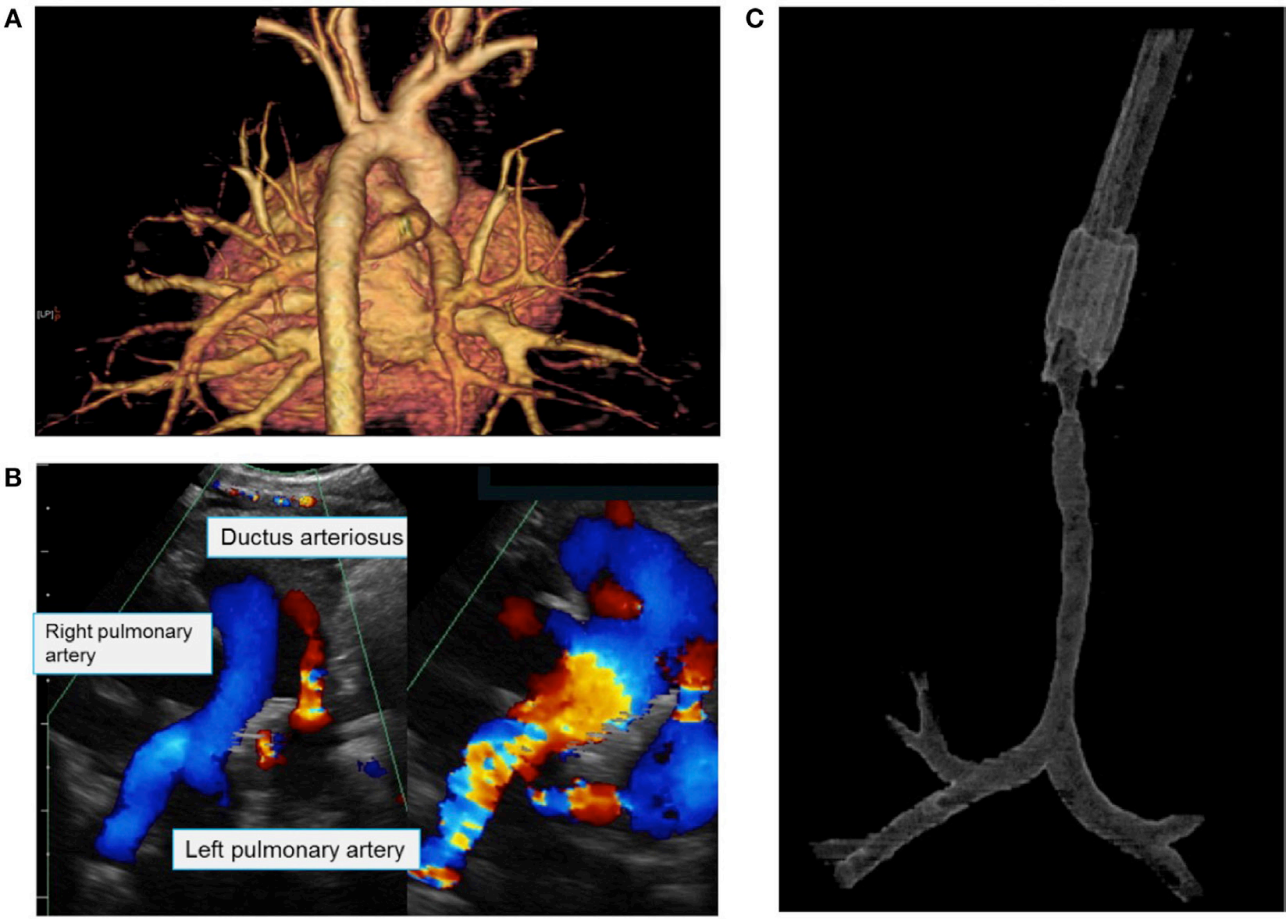
**(A)** 3D reconstruction of the great vessels based on a chest CT showing the diagnosis of pulmonary artery sling with aberrant origin of the left pulmonary artery (LPA) from the right pulmonary artery. **(B)** Echocardiography showing the main pulmonary artery, the right pulmonary artery and the LPA originating from the right pulmonary artery, as well as patent ductus arteriosus. **(C)** 3D reconstruction of the upper airways based on a chest CT showing long-segment tracheal stenosis beginning at the fifth tracheal ring and ending 5 mm above the carina.

**Figure 2:**
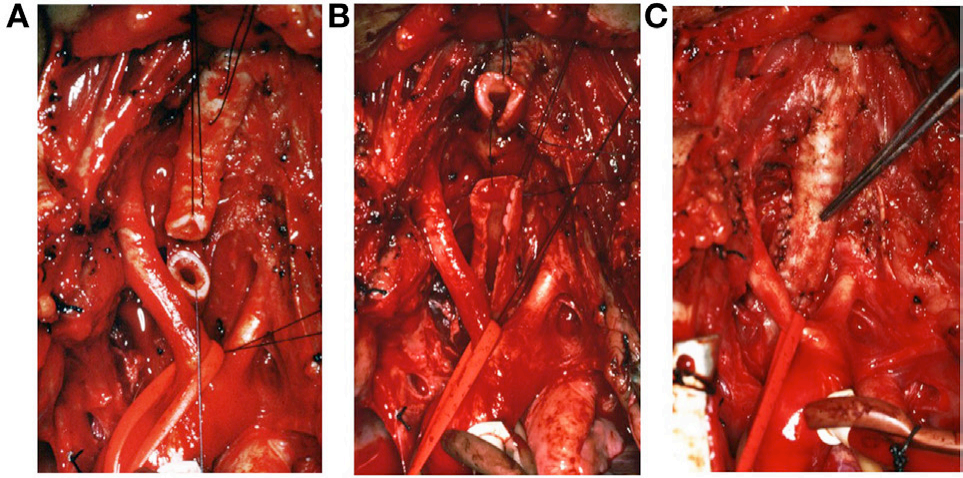
**Intraoperative view of the trachea and the slide tracheoplasty procedure**. A horizontal section of the stenotic trachea is performed **(A)**, followed by vertical incisions of the proximal and distal parts **(B)**, and finally elliptic anastomosis at the cut surface **(C)**. With this procedure, the tracheal length is reduced by half.

**Figure 3:**
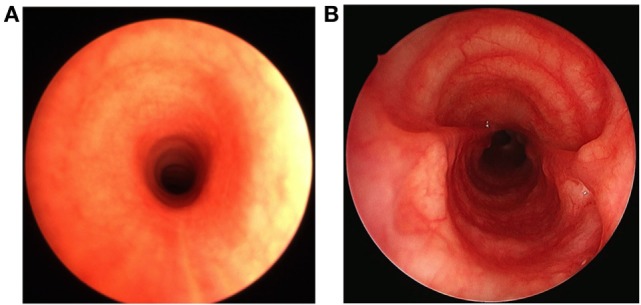
**Pre- and postoperative bronchoscopic views of the trachea**. Before the surgical intervention, the trachea shows a significantly reduced tracheal lumen due to long-segment tracheal stenosis **(A)**. After the slide tracheoplasty, the cross section of the tracheal lumen increases fourfold **(B)**.

A 7-week-old boy was transferred to our Institution after being hospitalized for 4 weeks at another University Hospital for RSV bronchiolitis necessitating mechanical ventilation. Because the respiratory situation was not improving, he underwent flexible bronchoscopy, chest CT, and echocardiography. The examinations revealed PAS associated with a PDA, an atrial septal defect, and a diffuse LSTS with eight complete cartilaginous rings extending from the fifth tracheal ring to five millimeters above the carina (Figure 1). Our Institution specializes in pediatric tracheal surgeries, therefore the surgical intervention was performed at our Institution. After doing a median sternotomy and putting the child on cardiopulmonary bypass, ligation then section of the PDA and LPA reimplantation were performed followed by slide tracheoplasty (Figures 2 and 3). Total concomitant cardiac and tracheal surgery duration was 317 minutes, of which 140 minutes were on cardiopulmonary bypass.

Respiratory and cardiovascular problems occurred after the operation. The major respiratory problem consisted of repetitive failure of weaning from mechanical ventilation. The first attempt was done on the eighth postoperative day (POD), but the patient presented severe respiratory distress and stridor despite non-invasive ventilation and was reintubated the same day. Endoscopy revealed left vocal cord paralysis and significant bronchomalacia of both stem bronchi, without any sign of anastomotic failure at the site of the tracheoplasty. The second extubation was performed on POD 18, again followed by non-invasive ventilation. Because of severe respiratory distress, the patient was reintubated 3 days later. Endoscopy of the airways showed the development of granulation tissue at the level of the anastomosis, which was removed. On POD 28, third extubation trial was done, but 2 days later, mechanical ventilation was again required. Endoscopy revealed a constriction of the right main stem bronchus requiring balloon dilatation. The patient was successfully weaned from mechanical ventilation on POD 45, with the need for non-invasive ventilation for another 2 months. Endoscopy 3 months after the operation showed a slight stenosis of the trachea and both main stem bronchi, responding each to balloon dilatation, persisting left vocal cord paralysis and persisting but improving bilateral bronchomalacia. Despite these findings, the patient clinically improved and could be transferred to the referring hospital without any respiratory support or oxygen therapy. The last endoscopic control 1 year after the surgery showed a satisfactory postoperative result without any sign of granulation tissue or stenosis of the airway. The left vocal cord was still paralyzed with optimal right vocal cord compensation, and there was mild distal tracheal and bronchomalacia.

**Figure 4:**
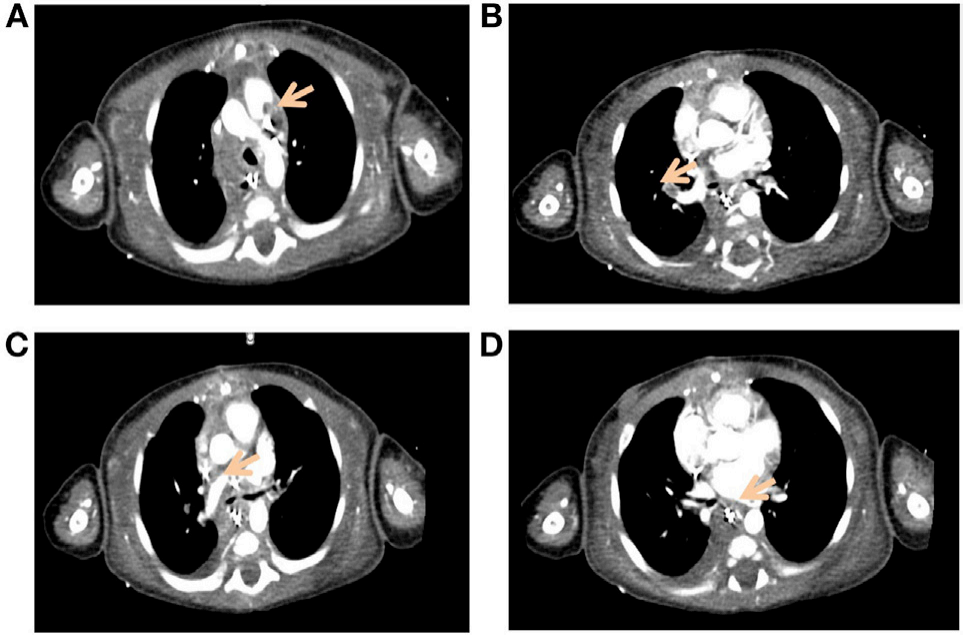
**Axial view of the pulmonary artery on angio-CT of the chest**. The images show bilateral pulmonary artery thrombosis (arrows), with thromboses of the main pulmonary artery **(A)**, of the upper branch of the right pulmonary artery **(B)**, and of the upper **(C)** and lower branches of the left pulmonary artery **(D)**.

From the cardiovascular point of view, the postoperative period was also challenging. In the context of worsening oxygenation on POD 10, echocardiography was performed showing absent LPA flow. CT scan revealed bilateral pulmonary artery thrombosis (Figure 4). Therapeutic anticoagulation with unfractionated heparin was started and maintained for 2.5 weeks. Response to treatment was good, and repermeabilization of the pulmonary arteries could be demonstrated after 48 hours.

Repeated echocardiograms showed patency of the LPA, but mild to moderate stenosis at the reimplantation site as well as discrete stenosis of the right pulmonary artery at the level of the initial origin of the LPA. Due to the persistence of these stenoses during the follow-up, percutaneous balloon dilatation was performed successfully one year after the surgery.

The total length of stay in hospital of this patient was 184 days, 24 days thereof preoperatively and 108 days in the pediatric intensive care unit, before he could be discharged home. The total duration of mechanical ventilation was 70 days, 25 days thereof preoperatively. On last follow-up, the patient is doing well without any respiratory symptoms. He has no bronchoaspiration while feeding, and the voice is mildly hoarse but communicable. Future follow-up appointments will assess long-term outcome and evaluate if this long hospitalization in early infancy will affect long-term neurologic development. Currently, the patient has a normal neurologic development.

## Discussion

Pulmonary artery sling, often associated with intrinsic tracheal stenosis, is a vascular malformation compressing the airway and leading to progressive respiratory symptoms. It is not rare, as in our case, that intubation and mechanical ventilation is required (5, 7, 8). Surgical management is curative and consists of reimplantation of the LPA and tracheoplasty. This surgery is nowadays associated with a low morbidity and mortality, especially when using the slide tracheoplasty technique (5, 9). This technique has been adopted in our Institution for almost 20 years and is described in a former article written by members of our Airway Unit (10).

Complications reported in the literature are mostly due to the tracheoplasty. In a study of 14 children who underwent slide tracheoplasty, with 9 of them having concomitant LPA reimplantation, the authors identified 2 children with postoperative complications. One of them developed granulation tissue and restenosis of the trachea requiring repeated balloon dilatations, placement of a stent, and finally surgical resection of the restenosis. The other child had anastomotic dehiscence and underwent a second slide tracheoplasty, later also developed restenosis requiring balloon dilatation and stent placement (6). Oshima et al. examined postoperative complications of 31 children undergoing PAS repair, with 28 of them also having slide tracheoplasty in a single stage. Five children developed granulation tissue, 3 children had tracheomalacia, and 2 had anastomotic leakage (8).

In our patient, most of the abovementioned complications occurred consecutively. Even though there were many adverse outcomes, these cannot be explained only by the surgery itself. The intervention was performed in a center that specializes in pediatric tracheal surgeries and that has a lot of experience in pediatric cardiac surgery. The duration of surgery as well as the time on cardiopulmonary bypass was well within those mentioned in the literature for this type of complex combined surgery (11, 12).

The repeated extubation failures can be explained by the following reasons. First, the child needed mechanical ventilation for bronchiolitis preoperatively, and it is a well known fact that patients requiring respiratory assistance before such a surgery have an increased risk for prolonged postoperative ventilation and increased morbidity (11). The viral infection that led to the intubation in the first place could also play a role as it increases pulmonary hypersensitivity that may have persisted after the surgery. It is difficult to know if the postoperative outcome could have been less complicated if we had waited longer for the lungs to recover before performing the surgery. However, we considered that the heart–lung status secondary to a severe tracheal stenosis left untreated could have worsened the postoperative period if the surgery was performed later. Finally, the association of a significant bronchomalacia along with the LSTS can explain the prolonged mechanical ventilation, repeated extubation failures, and the long duration of non-invasive ventilation. Repeated intubations and the prolonged presence of an endotracheal tube explain the development of granulation tissue. The majority of these cases are associated with varying degrees of bronchomalacia. Preoperative analysis of LSTS includes passing a small caliber endoscope through a “steadier” complete ring and non-collapsible stenosis. Hence, the knowledge of the degree of the distal airway collapse is not always possible or precise. One hypothesis explaining certain degrees of malacia in the repaired trachea could be related to the surgery itself which now weakens a complete ring. The typical postoperative stenosis can be related to the cartilage elasticity that has a tendency to curve on itself. These stenoses respond well to balloon dilatation and temporary stent placement. Extreme cases, require revision surgery.

The patient presented, in addition, a persistent left vocal cord paralysis. This complication is not yet described after PAS repair and slide tracheoplasty, but is known to occur after cardiac surgery, yet not necessarily related to the tracheal correction. In 1 retrospective review of 183 patients with aortic arch anomalies causing tracheoesophageal compression, thereof 8 children with PAS, 1 patient was found to have postoperative vocal cord paralysis (13). However, it is not mentioned if it was one of the children with PAS, and recurrent laryngeal nerve injury is more likely to occur after aortic arch rather than pulmonary artery surgery. Ligation of a PDA can also cause injury to the left recurrent laryngeal nerve with the result of left vocal cord paralysis (14). We believe that this was the case in our patient. A compromised glottis due to unilateral vocal cord paralysis could also contribute to the need for prolonged respiratory assistance. However, not all neonates or small infants with this complication need aggressive respiratory assistance. The growth of the larynx with age and the increased airway geometry spontaneously corrects this problem. The nerve and vocal cord recovery may take 6 months to several years (7).

Left pulmonary artery stenosis after the reimplantation is a well known though infrequent complication. Two studies with 21 and 31 patients, respectively, undergoing LPA reimplantation found each only one child to have severe LPA stenosis (5, 8). However, in an older report of 2005, 4 patients out of 14 children with PAS developed LPA stenosis after the surgical intervention (1). Echocardiographic studies in our patient also revealed this complication, which responded well to balloon dilatation.

In the early postoperative period, our patient developed bilateral pulmonary thrombosis at the anastomosis and suture sites of the pulmonary arteries with embolism to the smaller branches. To the best of our knowledge, this is the first case describing this event after LPA reimplantation. A possible explanation for the occurrence of these thrombi could be the turbulent blood flow due to distortion of the pulmonary arteries in the context of the LPA repair and the development of stenoses at the LPA reimplantation site and at the initial origin of the LPA from the right pulmonary artery, and the associated circular anastomoses of these vessels. Additionally, cardiopulmonary bypass causes an imbalance of the hemostatic system with a risk of both hemorrhagic and thrombotic complications (15). The patient described in this report did well under anticoagulation therapy, and repermeabilization of the pulmonary arteries was observed after 48 hours of treatment.

There are currently no guidelines regarding anticoagulation treatment of pulmonary artery thrombosis after cardiovascular surgery in children, or antithrombotic prophylaxis after PAS repair. As our patient responded well to unfractionated heparin and was monitored closely in the intensive care unit, we decided to discontinue the anticoagulation therapy after 2.5 weeks of treatment and demonstration of a complete resolution of the pulmonary artery thromboses. The patient did not present recurrent pulmonary artery thrombosis or any other thrombotic event. Therefore, we did not perform thrombophilia workup.

In conclusion, this case report illustrates that postoperative management after PAS repair and slide tracheoplasty can still be challenging, despite ongoing improvements in surgical technique and intensive care medicine. However, with appropriate management of the postoperative complications, the long-term outcome can still be favorable.

## Ethics Statement

The parents gave written informed consent to the publication of this case, and it was approved by our institutional Ethics Committee.

## Author Contributions

AW reviewed the patient’s chart and drafted the manuscript. BD was the pediatric cardiologist of the patient and helped to establish the diagnosis. She provided the images of the first CT scan and echocardiography of the patient. She critically revised the manuscript. M-HP was the attending physician at the pediatric intensive care unit during the long hospitalization of the patient at the University Hospital of Lausanne. She critically revised the manuscript. SB performed the percutaneous balloon dilatation of the left pulmonary artery in this patient. He critically revised the manuscript. DT performed the first endoscopy of the airways and was in charge during the patient’s stay in the University Children’s Hospital of Basel. He critically revised the manuscript. KS performed the slide tracheoplasty. He provided the images of the surgery and endoscopy. He contributed to the writing of the discussion and critically reviewed the manuscript. NS was the pediatric cardiologist in charge of the patient during his stay at the University Hospital of Lausanne. The manuscript was written under her supervision and she critically reviewed it. All authors approved the final version as submitted.

## Conflict of Interest Statement

The authors declare that the research was conducted in the absence of any commercial or financial relationships that could be construed as a potential conflict of interest.

## Abbreviations

PAS, pulmonary artery sling; LPA, left pulmonary artery; PDA, patent ductus arteriosus; LSTS, long-segment tracheal stenosis; POD, postoperative day.
